# Inverting hydrolases and their use in enantioconvergent biotransformations

**DOI:** 10.1016/j.tibtech.2013.05.005

**Published:** 2013-08

**Authors:** Markus Schober, Kurt Faber

**Affiliations:** Department of Chemistry, Organic and Bioorganic Chemistry, University of Graz, Heinrichstrasse 28, A-8010 Graz, Austria

**Keywords:** enantioconvergent process, inversion, retention, deracemization, epoxide hydrolase, sulfatase, dehalogenase

## Abstract

Owing to the more abundant occurrence of racemic compounds compared to prochiral or *meso* forms, most enantiomerically pure products are obtained via racemate resolution. This review summarizes (chemo)enzymatic enantioconvergent processes based on the use of hydrolytic enzymes, which are able to invert a stereocenter during catalysis that can overcome the 50%-yield limitation of kinetic resolution. Recent developments are presented in the fields of inverting or retaining sulfatases, epoxide hydrolases and dehalogenases, which allow the production of secondary alcohols or vicinal diols at a 100% theoretical yield from a racemate via enantioconvergent processes.

## Highlights

• Enantioconvergent processes overcome the 50%-yield limits of kinetic resolution.
• Inverting enzymes are key catalysts for enantioconvergent processes.
• Enzyme engineering provided improved variants of inverting enzymes.

## Single enantiomers from racemates

**Figure 1:**
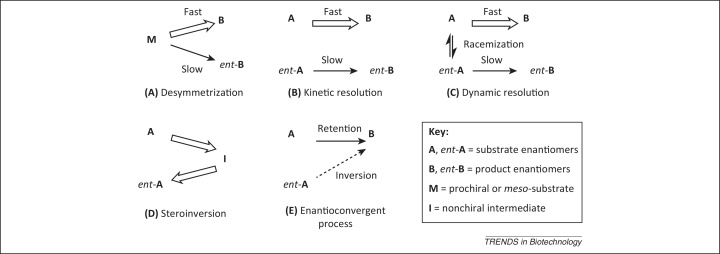
Strategies for the generation of single enantiomers from racemates in 100% theoretical yield. **(A)** Desymmetrization of a prochiral or *meso*-compound **M** producing **B** as sole product. **(B)** Kinetic resolution of a pair of enantiomers (**A** and *ent*-**A**) yielding **B** and nonreacted *ent*-**A**. **(C)** Dynamic resolution of a pair of enantiomers **A** and *ent*-**A** furnishing **B** as the sole product. **(D)** Stereoinversion of enantiomer **A** via nonchiral intermediate **I** to yield its mirror-image counterpart *ent*-**A** as the sole product. **(E)** Enantioconvergent transformation of enantiomers **A** and *ent*-**A** through opposite stereochemical pathways forming **B** as sole product.

Enzymes are gaining increasing attention as catalysts in the chemical and pharmaceutical industry 1, 2, 3, as they are the key to novel [4], more selective and ‘greener’ processes [5]. In the context of stereoselective (see Glossary) transformations, the trend favors processes that produce a single product at 100% theoretical yield without occurrence of an unwanted enantiomer, which has to be regarded as economic ballast (Figure 1). In a classic approach, desymmetrization of prochiral or *meso*-compounds (**M**) represents a potent strategy for achieving this 6, 7. However, most chiral products are obtained via racemate resolution owing to the more abundant occurrence of chiral compared to prochiral substrate molecules. In most cases, the residual 50% of unwanted substrate (*ent*-**A**) possessing the ‘wrong’ absolute configuration is not applicable for further processing, which sets a low economic ceiling in kinetic resolutions. This drawback can be overcome by three different methods.

**Figure 2:**
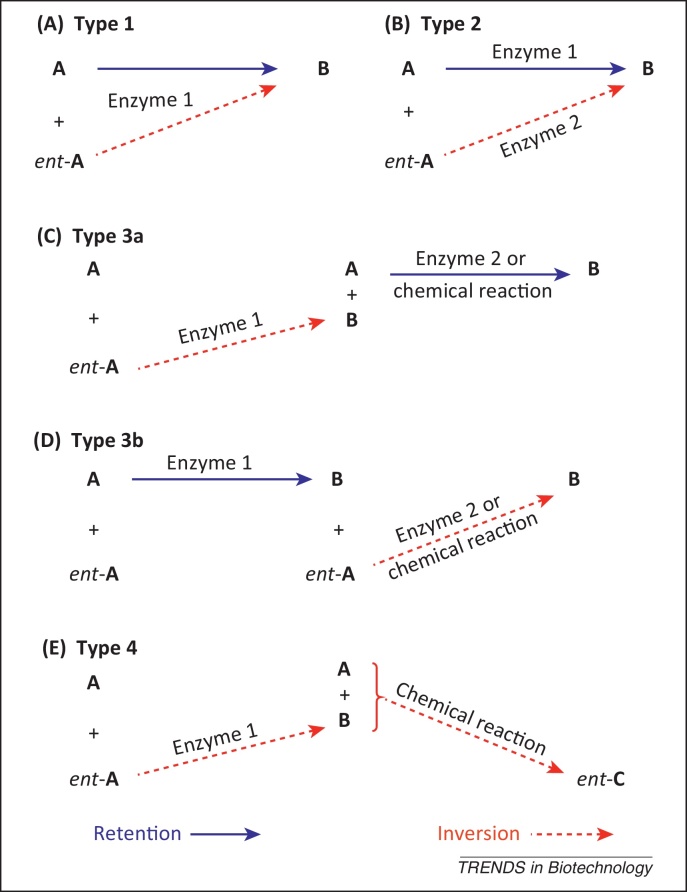
Classification of (chemo)enzymatic enantioconvergent processes. **(A,B)** Single-step enantioconvergence via a one-enzyme **(A)** or two-enzyme **(B)** process. **(C,D)** Two-step enantioconvergence via inversion/retention **(C)** or retention/inversion **(D)**. **(E)** Two-step enantioconvergence via inversion followed by chemical reaction. **A**, *ent*-**A** = substrate enantiomers; **B**, *ent*-**B** = product enantiomers; *ent*-**C** = final product enantiomer.

The first and most frequently applied technique is dynamic kinetic resolution 8, 9, 10, which is used for the deracemization of chiral alcohols, amines, amino acids, and carboxylic acids. These processes combine an (enzymatic) kinetic resolution with *in situ* racemization of substrate enantiomers **A** and *ent*-**A** by a chemocatalyst or biocatalyst. The second technique is stereoinversion 11, 12, in which one enantiomer (**A**) of a racemic mixture is selectively converted into an achiral intermediate (**I**) that is reconverted into the opposite enantiomer, *ent*-**A**. Multienzymatic [13], chemoenzymatic [9], and chemical systems [14] have been developed. This review is focused on the third technique, that is, an enantioconvergent process that makes use of two different reactions, which convert a pair of enantiomers, **A** and *ent*-**A**, via retention and inversion of configuration, respectively, to yield a single product enantiomer, **B**, with a 100% theoretical yield. In recent years, enzymatic 15, 16, chemoenzymatic 17, 18, 19, and chemical processes [20] have been developed. This review focuses on enzyme-based protocols, which can be classified into four types (Figure 2).

### Type-1

In a type-1 process, a single enzyme converts both substrate enantiomers (**A** and *ent*-**A**) through opposite pathways, that is, via inversion and retention of configuration to furnish **B** as the sole enantiomeric product via an elegant single-enzyme one-pot protocol. The requirements for the catalytic performance for such a (bio)catalyst are challenging, as the enzyme must not show any enantiopreference (by converting one enantiomer leaving the other untouched); rather, it should transform both enantiomers (**A** and *ent*-**A**) at comparable rates but through opposite stereochemical pathways. Such a process has so far only been realized for epoxide hydrolases 15, 21, 22, 23, 24.

### Type-2

If a single enzyme possessing the above requirements is not available, two proteins can be combined in a type-2 process. In this case, both enzymes must show opposite enantiopreference and a matching complementary stereoselectivity in terms of inversion or retention. When both enzymes show high enantioselectivity, they can act simultaneously on both substrate enantiomers (**A** and *ent*-**A**) in a one-pot protocol. Type-2 reactions have so far been shown for epoxide hydrolases 25, 26, 27 and alkyl sulfatases [16].

### Type-3

If one enzyme out of a matching stereocomplementary pair shows insufficient enantioselectivity, bi-enzymatic one-pot two-step processes of type 3 may be designed. In this case, the reaction is conducted to 50% conversion by an enantioselective enzyme via retention or inversion, to affect kinetic resolution yielding either a homochiral or heterochiral product mixture (**A** + **B** or *ent*-**A** + **B**, respectively). Then, in a second step, the remaining non-converted substrate enantiomer (**A** or *ent*-**A**) is reacted with matching opposite stereochemistry via retention or inversion of configuration. Because the second step starts with a single enantiomer **A** or *ent*-**A** (which was not converted in the preceding kinetic resolution step), enantioselectivity is not required. Examples of bi-enzymatic type-3 reactions are known for alkyl sulfatases [16]. Alternatively, chemocatalytic methods may be used for the second (nonenantioselective) step to furnish chemoenzymatic type 3 protocols. So far, no one-pot protocol has been developed owing to the incompatibility of the (harsh) reaction conditions of the chemical with those of the enzymatic step. Owing to the higher enantioselectivity of enzymes compared to chemical catalysts in general, the first step is invariably catalyzed by a protein, whereas the second step is mediated by a (nonenantioselective but stereoselective) chemocatalyst. Type-3a processes rely on an inverting enzyme and lead to a homochiral mixture of substrate **A** and product **B** after the first enzymatic step. Consequently, the remaining substrate **A** must then be converted with retention of configuration to furnish **B** as the sole product. This process has so far been developed for inverting alkyl sulfatases 16, 17. Owing to the more abundant occurrence of retaining enzymes, such as lipases, esterases, and proteases, several type-3b applications are known. In this case, the first step comprises a stereoselective retaining enzymatic reaction that leads to a heterochiral mixture of substrate *ent*-**A** and product **B**. In the second step, the residual substrate *ent*-**A** must be converted via an inverting chemical (or enzymatic) reaction to furnish **B** 16, 28, 29, 30, 31.

### Type-4

In type-4 reactions, an inverting enzyme is processing one substrate enantiomer, leading to a homochiral mixture of substrate **A** and product **B**, both of which are subjected to a subsequent chemical (substitution) reaction with inversion of configuration to furnish product *ent*-**C**. This process is favorable when both substrate **A** and product **B** have a strong leaving group that can easily be replaced. In some cases, a weak leaving group has to be converted into a better one via an additional activation step. This process has been verified for haloalkane dehalogenases [19].

This review illustrates the power of stereocomplementary hydrolases for the design of enantioconvergent bioprocesses. To date, most enzymes have been found to act via retention of configuration; inverting hydrolases occur more rarely and so far have only been identified within the groups of sulfatases, epoxide hydrolases, and dehalogenases. Although glycosidases may act through retention or inversion at the anomeric center of a carbohydrate substrate, they are not covered in this study because they solely act on diasteriomeric (rather than enantiomeric) substrates, which leads to de-epimerization as opposed to de-racemization [32].

## Sulfatases

**Figure 3:**
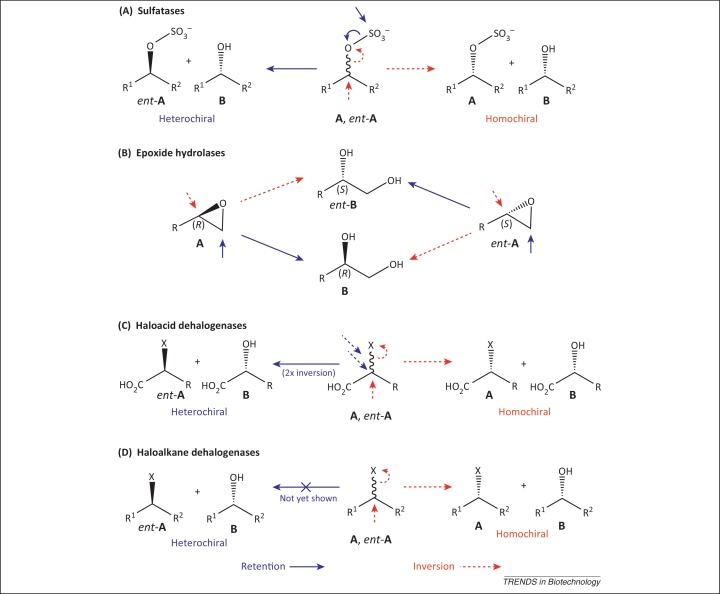
Stereochemical action of inverting and retaining hydrolytic enzymes. **(A)** Retaining and inverting sulfatases act via breakage of S–O and C–O bonds, respectively. **(B)** Inverting and retaining epoxide hydrolases act through regio-complementary attack at substituted or non-substituted oxirane carbon atoms, respectively. **(C)** Inverting and retaining haloacid dehalogenases act via single or double S_N_2-displacement of halide, respectively. **(D)** Inverting haloalkane dehalogenases act via S_N_2-displacement of halide, whereas the mode of action of retaining haloalkane dehalogenases is unknown.

In addition to their application in the biodegradation of sulfur-containing organic waste and their role in various cellular functions [33], sulfatases have intensively been studied for their application in the deracemization of *sec*-alcohols 16, 34, 35. They have been classified into three groups according to their mechanism (Figure 3) [36].

Arylsulfatases are the best-studied group. These enzymes contain the hydrate form of a formyl glycine residue (derived via post-translational modification of serine or cysteine), which triggers a nucleophilic attack on sulfur. This leads to a cleavage of the CO–S bond of the sulfate ester [33], which in turn leads to retention of configuration at the chiral carbon center. The second group consists of Fe(II) α-ketoglutarate-dependent alkylsulfatases that belong to the dioxygenase superfamily [37]. They oxidatively convert *prim*-sulfate esters into the corresponding aldehyde and inorganic sulfate, which results in the destruction of a chiral center. The third group of sulfatases is formed by the metallo β-lactamase-related Zn^2+^-dependent alkylsulfatases. The active site of these enzymes contains a binuclear Zn^2+^ cluster that activates a water molecule, which triggers a nucleophilic attack on the chiral carbon center. In contrast to aryl sulfatases, this leads to a breakage of the C–OS bond, causing inversion of configuration at the chiral carbon atom. Sulfatase activity in various microorganisms on *sec*-alkyl sulfates proceeding via inversion 35, 38 and retention [39] has been found, but members of both groups of sulfatases were only recently recombinantly produced and characterized on a molecular level 16, 17. The first structure of a group III sulfatase (SdsA1 from *Pseudomonas aeruginosa*) was solved in 2006 [36]. This enzyme was found to be unsuitable for stereoselective sulfate ester hydrolysis because it strongly prefers nonchiral *prim*- over *sec*-alkyl sulfates 17, 40. The first highly selective inverting *sec*-alkyl sulfatase was Pisa1 from *Pseudomonas* DSM 6611, the structure of which has recently been elucidated [40]. The reaction rates for Pisa1 were much higher than for biotransformations with whole cells of this organism [38] and reached the critical 50% threshold for many substrates in kinetic resolution experiments. Inversion of configuration was proven by reactions with enantiopure substrates and, in addition, by labeling studies, which showed exclusive incorporation of ^18^O in the product alcohol using ^18^O-labeled buffer [17]. Detailed investigation of the substrate spectrum of Pisa1 [41] revealed excellent stereoselectivities and perfect 50% conversions for various (ω-1)-, (ω-2)- and 1-alkyn-3-yl sulfates. Terminal allylic substrates showed lower enantioselectivity in comparison to propargylic counterparts owing to competing (nonenzymatic) autohydrolysis. Competing autohydrolysis could be significantly reduced by addition of water-soluble cosolvents such as dimethyl sulfoxide (DMSO). In addition, benzylic *sec*-alkylsulfate esters, which also showed severe autohydrolysis, could be stabilized by the presence of electron-withdrawing groups on the aromatic ring (M. Toesch et al., unpublished). A chemoenzymatic deracemization protocol requiring medium change (Figure 2, type 3a) using Pisa1 (in the first step) and acidic hydrolysis (*p*-toluenesulfonic acid, MTBE/H_2_O 98:2) in the second step, could be developed (Table 1) [16]. This protocol was successfully applied to 5-hexen-2-yl sulfate as the key step in the total asymmetric synthesis of (*R*)-lasiodiplodin methyl ether, a precursor of the anti-leukemic agent lasiodiplodin [42]. An analogous one-pot two-step protocol was developed for 2-octyl sulfate [41], but its applicability was limited by the strongly acidic reaction conditions. These drawbacks could be circumvented by the discovery of retaining *sec*-alkyl sulfatase activity for *P. aeruginosa* arylsulfatase (PAS) [16]. Because the enzyme showed opposite enantiopreference compared with Pisa1, both enzymes could be combined in three different types of one-pot enantioconvergent processes of type 3: In the first case, highly enantioselective inverting Pisa1 yielded a homochiral mixture of formed alcohol and nonconverted sulfate via kinetic resolution. The residual substrate was hydrolyzed by PAS with retention of configuration (Figure 2, type 3a). In cases in which PAS showed high enantioselectivity but Pisa1 did not, both enzymes were used in reverse order, that is, PAS was applied first, leading to a heterochiral mixture. The remaining sulfate was then converted by Pisa1 with inversion of configuration (Figure 2, type 3b). When both enzymes were enantioselective, an elegant one-step protocol could be developed (Figure 2, type 2). Although a one-enzyme one-pot process of type 1, as shown for epoxide hydrolases 15, 21, is theoretically possible (Figure 2, type 1), the likelihood for its realization is low, as it would require a single sulfatase acting via nucleophilic attack at S versus C on both substrate enantiomers. Nucleophilic attack at S versus C is a consequence of the enzyme mechanism, which completely differs for both types of sulfatases.

**Table 1:** Deracemization reactions using sulfatases, epoxide hydrolases, and dehalogenases^a^

Substrate	Product	R	Inversion	Retention	Absolute configuration product	Selectivity^b^	Conversion^c^	Process type	Refs
***Sulfatases***									
		*n*-alkyl (C_4_-_8_) (CH_2_)_2_-CH=CMe_2_ (CH_2_)_2_CH=CH_2_ (CH_2_)_1,2_Ph, *c*-C_6_H_11_	Pisa1	Acid catalysis	(*S*)	+++	+++	3a	17, 41
		*n*-alkyl (C_4,5_)	Pisa1	Acid catalysis	(*S*)	+++	+++	3a	16, 41
		*n*-alkyl (C_5_-_7_)	Pisa1	Acid catalysis	(*R*)	+++^d^	+++	3a	[41]
		*n*-alkyl (C_4,5_) CH_2_-CHMe_2_	Pisa1 Pisa1	PAS Acid catalysis	(*R*) (*R*)	+++ +++	+++ +++	3a	16, 41
		Me, Et	Pisa1	PAS	(*S*)	+++	+++	3b	[16]
		–	Pisa1 Pisa1	PAS Acid catalysis	(*S*) (*S*)	+++^d^ +++^d^	+++ +++	2 3a	16, 41
		*m*-CF_3_, *m*,*m*-(CF_3_)_2_	Pisa1 Pisa1	PAS Acid catalysis	(*S*) *(S)*	+++^e^ +++^e^	++ +++	2 KR	16, 41
***Epoxide hydrolases***									
		H, *m*-, *p*-Cl	CcEHase BsEHase	CcEHase AnEHase	(*R*) (*R*)	++ ++	+++ +++	1 2	24, 43 27, 43
		*n*-C_5_H_11_, Ph, (CH_2_)_3_CH=CH_2_, (CH_2_)_4_Br, CH_2_OBn	Acid catalysis	Retaining EHase	(*S*)	+++	+++	3b	43, 97
		R^1^: *n*-alkyl (C_1,2,4,8_) Cl-CH_2_, HO-(CH_2_)_2,10_, (CH_2_)_7_CO_2_H, CH_2_CH=CH(CH_2_)_7_CO_2_H R^2^: *n*-alkyl (C_3-5,7,10_), (CH_2_)_7_CO_2_H, (CH_2_)_10_OH, CH_2_CH=CH-C_5_H_11_	Inverting EHase	n.a.	(*R*,*R*)	++	+++	1	[43]
***Haloacid dehalogenases***									
		–	L-α-HADH (*P. putida*)	Base catalysis	(*R*)	++	n.d.	3a^f^	78, 79
***Haloalkane dehalogenases***									
		*n*-alkyl (C_3,4_)	DatA DbjA	n.a.	n.d. (*S*)	+++ ++	+++ +++	KR	[85] 86, 87
		Me, Et	DatA DbjA DhaA LinB	n.a.	n.d. (*S*) n.d. n.d.	+++ +++ +++ +	+++ +++ +++ +++	KR	[85] 86, 87 [86] [86]
		Me, Et	Dbja DhaA DpcA LinB	n.a.	(*S*) n.d. (*S*) n.d.	+++ +++ +++ +++	+++ +++ n.d. +++	KR	86, 87 [86] [98] [86]
		Ph, Bn, CH_2_CO_2_*t*-Bu	DbjA DhaA DhA31	Nu	(*S*) (*S*) (*S*)	+++ +++ +++	+++ ± +++	4 KR KR	19, 93 [93] [93]
		Bn	LinB	Nu	(*S*)	+++	+	4	19, 93
		R^1^: CH_2_CO_2_*t*-Bu R^2^: Me, Bn	LinB	n.a.	(*S,S*)^g^	+++	+++	4	[93]
		–	R5-90R R5-97S	n.a.	(*R*) (*S*)	++ +++	n.d. n.d.	DS	[91]

a Abbreviations: An., *Aspergillus niger*; Bn, benzyl = CH_2_Ph; Bs, *Bacillus subtilis*; Bu, butyl; Cc, *Caulobacter crescentus*; DS, desymmetrization of prochiral substrate; EHase, epoxide hydrolase; Et, ethyl; HADH, haloacid dehalogenase; KR, kinetic resolution; PAS, *P. aeruginosa* arylsulfatase; Ph, phenyl; n.a., not applicable; n.d., not determined; Nu, nucleophile.

b Enantioselectivity of kinetic resolution with inverting enzyme: +++, E > 200; ++, 200 > E > 100; +, 100 > E > 50; ±, 50 > E > 25; –, E < 25. Enantiomeric excess of product from enantioconvergent process: +++, >97%; ++, 97–90%; + 90–80%; ±, 80–60%; – <60%.

c Conversion in kinetic resolution with inverting enzyme: +++, >45%; ++, 45–40%; +, 40–30%; ±, 30–15%; –, <15%. Overall conversions of enantioconvergent process: +++, >90%; ++, 90–80%; +, 80–60%; ±, 60–30%; –, <30%.

d Competing autohydrolysis was suppressed by addition of 20% DMSO (v/v).

e High selectivity only in presence of electron withdrawing groups (R) on the aromatic ring.

f Described as enantioconvergent process, which seems to be impossible under the reaction conditions chosen.

g Owing to the presence of a second chiral center, a diastereomeric product is obtained.

Processes based on sulfatases have a high potential for large-scale applications, as substrate and product are easily separable by simple extraction owing to their different polarity.

## Epoxide hydrolases

Epoxide hydrolases have gained a lot of attention for their ability to produce enantiopure vicinal diols and epoxides, which are both important building blocks for the asymmetric synthesis of bioactive compounds [43]. Most of these enzymes belong to the α/β-hydrolase superfamily and act via nucleophilic S_N_2 attack of an aspartate residue that forms a transient covalent enzyme–substrate–ester intermediate with the substrate [44]. In the second step, an activated water molecule attacks the carbonyl moiety of the ester–intermediate, thereby releasing the product diol. Overall, this leads to inversion of configuration at the oxirane carbon atom being attacked (Figure 3). By contrast, few enzymes have been shown to act through a one-step mechanism via a borderline S_N_2 mechanism. The S_N_2 mechanism involves a direct attack of an activated water molecule at one of the epoxide carbon atoms, which is supported by general acid catalysis at the oxirane O-atom [45]. Because the structure and function of epoxide hydrolases has been reviewed [46] and the application of these enzymes in organic synthesis was covered in [43], this review focuses on recent advances in enzyme engineering of epoxide hydrolases and their application in enantioconvergent biotransformations.

Epoxide hydrolases are so far the only enzymes known that are able to achieve a one-enzyme enantioconvergent process (Figure 2, type 1). The reason is that epoxide hydrolases not only show enantioselectivity (by preferring one substrate enantiomer over the other) but also show regioselectivity with respect to which oxirane carbon atom is attacked (Figure 3). With epoxide hydrolases, nucleophilic attack is possible on two adjacent epoxide carbon atoms via the same enzyme mechanism, which is not possible with stereocomplementary *sec*-alkylsulfatases. Nevertheless, only few enantioconvergent processes using overexpressed epoxide hydrolases have been reported with high stereoselectivity and conversion (Table 1). Eight racemic styrene oxide derivatives have been tested with lyophilized whole cells of an overexpressed epoxide hydrolase from *Caulobacter crescentus* [21]. Best results were obtained for *p*-chlorostyrene oxide yielding the corresponding (*R*)-diol with 95% enantiomeric excess (ee) and 72% yield in a preparative-scale experiment. Metagenomic studies also proved to be a valuable source for novel epoxide hydrolases. The epoxide hydrolase Kau2 derived from biofilter DNA was able to convert racemic *cis*-1-phenyl-1,2-epoxypropane to its corresponding (1*R*,2*R*)-diol with 97% yield and >98% ee [47]. An epoxide hydrolase from *Aspergillus niger* was evolved by iterative combinatorial active-site saturation test (CAST), leading to significantly increased stereoselectivity (enantiomeric ratio E from 4.6 to 115) for glycidyl phenyl ether [48]. Every evolutionary step was analyzed by molecular dynamic simulations and molecular modeling, which provided insights into the enzymatic mechanism. The same enzyme was further engineered for the enantioconvergent hydrolysis of 2,3-disubstituted epoxides [49]. Several mutants yielded the corresponding *vic*-diol in 99% ee and >90% conversions. The same group was also able to broaden the substrate spectrum of limonene epoxide hydrolase from *Rhodococcus erythropolis* DCL 14 via iterative site directed mutagenesis (ISM) [50]. ISM was also used to alter the enantiopreference of potato epoxide hydrolase StEH1 for (2,3-epoxypropyl)benzene [51]. Further studies resulted in mutants with altered regio- and enantioselectivity [52]. Enantioselectivity enhancement by single-point mutation could be achieved in the substrate access tunnel of *A. niger* M200 epoxide hydrolase [53] and in the active site of *Agrobacterium radiobacter* epoxide hydrolase [54].

One-pot processes with two enantiocomplementary epoxide hydrolases (Figure 2, type 2) have recently been developed for the production of (*R*)-phenyl-1,2-ethanediol using enzyme pairs from *A. niger* LK/*Rhodotorula glutinis* [25] and *C. crescentus*/*Mugil cephalus* [26] with >90% ee and conversion. High selectivity and conversion were also obtained when two enzymes from the Neocarzinostatin gene cluster were combined in a biocatalytic deracemization protocol [55]. The main issue to solve is the evolution of epoxide hydrolases towards a broader substrate spectrum and enhanced stereoselectivities, next to improved activities and stability towards increased substrate concentrations.

## Dehalogenases

Dehalogenases are highly attractive enzymes for industry because they can be used not only for the bioremediation of recalcitrant halo-organic compounds [56] but also as catalysts for the synthesis of enantiopure building blocks [57]. This review focuses on the synthesis of enantiopure building blocks and covers recent advances and novel processes involving haloacid and haloalkane dehalogenases.

### Haloacid dehalogenases

Haloacid dehalogenases catalyze the hydrolytic dehalogenation of α-haloalkanoic acids to the corresponding α-hydroxyalkanoic acids (Figure 3) [58]. They have been classified either according to their sequence [59] or based on their stereoselectivity 60, 61; in this review the latter classification is used.

The best-studied group are l-2-haloacid dehalogenases that convert (*S*)-2-haloalkanoic acids into (*R*)-2-hydroxyalkanoic acids with inversion of configuration (Table 1). Several of their structures have already been solved and characterized 62, 63, 64, 65, 66. The mechanism proceeds via nucleophilic attack of an aspartate moiety on the chiral carbon center bearing the halogen, leading to a covalent enzyme–substrate–ester intermediate, which has a strong resemblance to that of S_N_2-type epoxide hydrolases. The ester-intermediate is then attacked by an activated water molecule, thereby triggering the product release. This mechanism has been supported by ^18^O-labeling experiments for the haloacid dehalogenase of *Pseudomonas* sp. YL [67]. Product labeling was found in multi- but not in single-turnover experiments, which suggests that the oxygen atom derived from water is incorporated into the nucleophilic Asp moiety via H_2_O attack on the enzyme–substrate–ester intermediate. The importance of this residue for catalysis was further strengthened by site directed mutagenesis 63, 68.

The second group are inverting dl-2-haloacid dehalogenases, which have been covered in a recent review [69]. They can convert both substrate enantiomers, which is quite unusual for enzymatic reactions and is only typical for the group of racemases [70]. Their reaction mechanism differs from l-2-haloacid dehalogenases such that it does not involve the formation of a covalent enzyme–substrate–ester intermediate, but proceeds through direct nucleophilic attack of an activated water molecule at the chiral carbon center. In this respect, it shows strong similarities with borderline-S_N_2-epoxide hydrolases. ^18^O-labeling studies showed that incorporation of ^18^O took place in both single- and multi-turnover reactions and no ^18^O-labeled enzyme fragments were found [71]. Detailed mechanistic studies on the structure of DheI from *Pseudomonas putida* PP3 [66] revealed that Asp189 was responsible for water activation, which is assisted by an adjacent Asn114 residue. Remarkably, dl-2-haloacid dehalogenase DL-DEX 312 from *P. putida* PP3 also acted on 2-chloro- and 2-bromopropionamide [72].

The third group of α-haloacid dehalogenases are inverting d-2-haloacid dehalogenases, which have the same catalytic mechanism (with high sequence similarity) as dl-2-haloacid dehalogenases but specifically act on the (*R*)-enantiomer of α-haloacids. Although their mechanism has been investigated [73], no crystal structure is yet available. Recently, structure prediction, molecular dynamics simulation and substrate docking were conducted for DheI from *Rhizobium* sp. RC1 to elucidate the catalytic residues [74]. Recently, literature on d-specific haloacid dehalogenases has been summarized [75].

The fourth group of α-haloacid dehalogenases are retaining dl-2-haloacid dehalogenases. So far, only one enzyme from *P. putida* PP3 was found to display this activity [76]. A mechanism involving double inversion of configuration was proposed but has not been proven, which is further complicated by the lack of sequence information.

The first stereoselective transformations involving α-haloacid dehalogenases were conducted with *rac*-α-chloropropionic acid (*rac*-CPA), the (*S*)-enantiomer of which was selectively hydrolyzed to (*R*)-lactic acid by an l-selective dehalogenase (Table 1). The residual non-converted mirror-image (*R*)-enantiomer was separated and was converted into (*S*)-lactate with a dl-2-haloacid dehalogenase, which overall constitutes kinetic resolution [77]. An industrial application of kinetic resolution of *rac*-CPA by an inverting (*R*)-haloacid dehalogenase was developed by AstraZeneca, leading to enantiopure (*S*)-CPA in >90% conversion. [78]. This process was also adapted to several other chiral short-chain α-haloacids and was shown to be suitable for a scale-up to >1000 t per annum. A chemoenzymatic enantioconvergent process (Figure 2, type 3a) for the production of (*R*)-lactic acid in 94% ee was claimed by combining an enantioselective inverting l-2-haloacid dehalogenase with base-catalyzed (chemical) hydrolysis of the residual substrate enantiomer with retention of configuration [79]. However, the latter step is known to proceed with S_N_2 inversion of configuration, which raises some doubts on the feasibility of this process. Furthermore, it has been stated [78] that CPA tends to racemize under basic conditions; this is likely to account for the high conversion beyond 50% in the first (enzymatic) step, which renders a dynamic resolution (rather than an enantioconvergent process), thereby making the second (chemical) step dispensable. In addition, the applicability of this process would be limited because it requires several separation steps in downstream processing owing to the incompatibility of the enzymatic step with the increased reaction temperatures and extreme pH required for the chemical hydrolysis. The ability of an l-2-haloacid dehalogenase to act in organic solvents [80] enabled the extension of the substrate spectrum to α-haloacids bearing lipophilic aromatic (phenyl and benzyl) and long alkyl (C_5_–C_16_) side chains.

The application of α-haloacid dehalogenases in deracemization processes has so far been limited to the production of enantiopure (*R*)-lactic acid using inverting enzymes, even though studies in the 1980s and 1990s indicated a much wider potential. The molecular characterization of an α-haloacid dehalogenase with retaining stereocomplementary would be a huge step towards the development of type 2 or 3 enantioconvergent processes for the production of enantiopure α-hydroxyacids from the corresponding easily accessible *rac*-α-haloacids. If more enzymes could be shown to be compatible with organic solvents, such as α-haloacid dehalogenase from *P. putida* [80], access to a wider substrate range would be opened.

### Haloalkane dehalogenases

In contrast to α-haloacid dehalogenases, which nucleophilically displace electronically activated halogen atoms, haloalkane dehalogenases produce alcohols from non-activated organohalogen compounds (Figure 2). This unique ability is the reason for their high potential in several areas of biotechnology, such as biocatalysis, by-product recycling, bioremediation, biosensing, cell imaging, and protein analysis, which were recently covered in a review [81]. Here, we delve into their potential and application in biocatalytic deracemization processes.

Haloalkane dehalogenases belong to the α/β-hydrolase superfamily. They hydrolytically convert halo-aliphatic substrates via a nucleophilic S_N_2-substitution into the corresponding alcohols with inversion of configuration at the chiral carbon center. Unlike the mechanistically related fluoroacetate and l-α-haloacid dehalogenases, they are not able to cleave carbon-fluorine bonds [82]. Their active site is located in a hydrophobic cavity between the α/β-hydrolase core and a variable cap domain. Access to the active site is regulated by a tunnel. The active site consists of a catalytic pentad with three key residues that are conserved amongst all enzymes – Asp acting as a nucleophile, His activating a water molecule, and Trp for halide leaving group stabilization. Haloalkane dehalogenases have been divided into three phylogenetic groups on the basis of sequence and structural data [83]. A more recent classification based on their substrate spectrum divides them into four substrate-specificity groups [84]. Besides mechanistic aspects, the enantioselectivity of haloalkane dehalogenases is of great importance, because the stereoselective nucleophilic displacement of a halide by [OH^−^] has no counterpart in traditional chemical methodology. An overview of highly selective enzymatic dehalogenation reactions is given in Table 1.

For simple *n*-2-haloalkanes, the selectivity is highest for 2-bromopentane and 2-bromohexane (DatA from *Agrobacterium tumefaciens*: E > 200 [85]) but decreases significantly (E < 30 for all tested enzymes) with increasing and decreasing chain length. By contrast, improved enantioselectivities were found on α-haloesters. This phenomenon was studied in more detail for DbjA from *Bradyrhizobium japonicum* USDA 110 using 2-bromopentane and methyl 2-bromobutyrate as substrates [86], which revealed different binding modes for the two substrate types. The stronger enantiomer discrimination for methyl 2-bromobutyrate is due to additional hydrogen bonding of its carbonyl group. Furthermore, it was shown that selectivity changes for both substrates were unrelated to each other when mutations in the surface loop area were performed [87]. Although pH changes led to a change in the oligomeric state of the enzyme, neither the secondary and tertiary structure nor the enantioselectivity was altered. By contrast, a temperature increase from 20 °C to 50 °C led to a significantly reduced E value (E from 174 to 13 for 2-bromopentane, E from 474 to 197 for ethyl 2-bromopropionate, and E from 225 to 83 for methyl 2-bromobutyrate). In a recent study, the thermostability and resistance to organic co-solvents of DhaA from *Rhodococcus rhodochrous* NCIMB13064 could be drastically improved, as proven by an increase of melting temperature T_M_ up to 19 °C and an extended half-life in 40% DMSO from minutes to several weeks [88]. Modification of the substrate tunnels proved to be a potent tool for obtaining highly improved dehalogenase variants. For instance, the catalytic activity on the toxic anthropogenic compound 1,2,3-trichloropropane (TCP) could be enhanced 32-fold and the efficiency by 26-fold [89]. In addition, the most active mutant, DhaA31 (which showed high activity but poor stereoselectivity), was further evolved towards the stereoselective conversion of TCP into (*R*)- or (*S*)-2,3-dichloropropan-1-ol, which can be chemically processed into enantiopure epichlorohydrin under basic conditions with inversion of configuration at their chiral center 90, 91. Carefully designed mutants giving (*R*)-2,3-dichloropropan-1-ol in 90% ee and the (*S*)-enantiomer in 97% ee could be obtained from a screening of only 5500 variants after five rounds of evolution. Recently, a one-enzyme tandem-desymmetrization/kinetic resolution process of short-chain prochiral and *meso*-dihaloalkanes was shown [92]. In this protocol, the dihaloalkane was converted to the haloalcohol in a first step, which was further dehalogenated to the corresponding diol. A highly enantioenriched haloalcohol was obtained when the slower-produced haloalcohol enantiomer was processed faster to the diol. By this means, the ee of (*R*)-3-bromo-2-methylpropan-1-ol could be pushed to 97% (24% conversion) and that of (*S*)-3-bromo-2-phenylpropan-1-ol (52% conversion) to 98%. Haloalkane dehalogenases were also applied to the preparation of α-hydroxyamides via deracemization. Fourteen α-bromoamides were subjected to kinetic resolution with five heterologously expressed haloalkane dehalogenases [93], and E values of >200 were achieved for several α-haloamides. Furthermore, molecular dynamic simulations with LinB–substrate complexes showed that the preferred (*R*)-enantiomer was more favorably bound in the active site. A dynamic kinetic resolution (DKR) procedure using polymer-based phosphonium bromide as the racemization catalyst led to highly enantioenriched α-hydroxyamides [94]. The reactions had to be conducted in a membrane reaction system to avoid inactivation of the enzyme by the racemizing polymer. The protocol is of high interest because it presents a DKR procedure that is not dependent on environmentally questionable heavy metal complexes. The first enantioconvergent process (Figure 2, type 4) based on haloalkane dehalogenases was recently developed for the chemoenzymatic synthesis of α-substituted amides, which are key intermediates in the production of pharmacologically active compounds [19]. In the first enzymatic kinetic resolution step, a homochiral mixture of α-bromo- and α-hydroxyamide was obtained using an inverting dehalogenase. In the second step, the formed α-hydroxyamide was chemically activated by a methylsulfonyl leaving group, which was subsequently displaced *in situ* by N, O and S nucleophiles, leading to highly enantioenriched α-substituted amides with excellent yields and selectivity. In summary, haloalkane dehalogenases possess a largely underestimated potential for the chemical industry, as they are able to not only degenerate toxic xenobiotic organohalogen compounds 95, 96 but also selectively convert them into useful chiral building blocks. For such transformations, counterparts in traditional chemical methodology occur rarely.

## Concluding remarks

Hydrolytic enzymes, such as sulfatases, epoxide hydrolases, and dehalogenases, possess the rare feature to act through different mechanisms affecting retention or inversion of configuration of a chiral carbon atom. Therefore, they represent crucial tools for the development of deracemization techniques, which allow the conversion of a racemic substrate mixture to a single enantiomeric product via independent enantioconvergent pathways with a 100% theoretical yield. To date, several successful applications have been shown for alkyl sulfatases, epoxide hydrolases, and dehalogenases. The crucial challenge for future developments is the extension of the number of inverting hydrolases, which may be achieved by the identification of novel enzymes from unexpected biodegradation pathways or by the re-engineering of the catalytic mechanism of well-known retaining hydrolases (Box 1).

Box 1 **Outstanding challenges**

• The search for retaining sulfatases that possess a broad substrate spectrum.
• The verification of retaining haloacid dehalogenases.
• The identification of retaining haloalkane dehalogenases.

## Glossary

Enantioconvergent process: two substrate enantiomers of a racemate are (simultaneously or step-wise) transformed via independent pathways through retention and inversion of configuration, respectively, yielding a single stereoisomeric product in 100% theoretical yield.
Enantioselectivity: preference for one enantiomer of a racemic chiral molecule in a (bio)chemical reaction.
E value (enantiomeric ratio): value describing the enantioselectivity of a kinetic resolution as the ratio of the reaction rate of substrate enantiomers. The latter can be calculated from *k*_cat_ and *K*_M_ values of enantiomers or from data sets of conversion (c) and enantiomeric excess of substrate (ee_S_) or product (ee_P_) when two out of the three parameters are known.
Heterochiral: compounds possessing an opposite sense of chirality.
Homochiral: compounds possessing the same sense of chirality.
Inversion: stereochemical switch of absolute configuration of a chiral carbon atom caused by S_N_2 reaction, hence substrate and product possess opposite absolute configurations.
Kinetic resolution: separation of enantiomers in a chemical reaction owing to different reaction rates of enantiomers. Enantiopure product and (non-reacted) substrate each can be obtained in 50% theoretical yields.
Retention: the absolute configuration of a chiral carbon atom is not altered during catalysis, hence substrate and product possess the same absolute configuration. Formal retention can also be obtained via subsequent double inversion.
Stereoselective: stereoselectivity describes the predominant formation of a stereoisomer in a chemical reaction: for instance, via retention or inversion of configuration.
